# The Global Rise of ESBL-Producing *Escherichia coli* in the Livestock Sector: A Five-Year Overview

**DOI:** 10.3390/ani14172490

**Published:** 2024-08-27

**Authors:** Antonio Mandujano-Hernández, Ana Verónica Martínez-Vázquez, Alma D. Paz-González, Verónica Herrera-Mayorga, Mario Sánchez-Sánchez, Edgar E. Lara-Ramírez, Karina Vázquez, Erick de Jesús de Luna-Santillana, Virgilio Bocanegra-García, Gildardo Rivera

**Affiliations:** 1 Centro de Biotecnología Genómica, Instituto Politécnico Nacional, Reynosa 88710, Mexico; jmandujanoh1800@alumno.ipn.mx (A.M.-H.); apazg@ipn.mx (A.D.P.-G.); elarar@ipn.mx (E.E.L.-R.); edeluna@ipn.mx (E.d.J.d.L.-S.); vbocanegg@hotmail.com (V.B.-G.); 2 Unidad Académica Multidisciplinaria Mante, Universidad Autónoma de Tamaulipas, Mante 89840, Mexico; evherrera@docentes.uat.edu.mx; 3 Laboratorio de Fisiología Vegetal, Centro de Investigación en Alimentación y Desarrollo A.C., Hermosillo 83304, Mexico; 4 Facultad de Medicina y Veterinaria Zootecnia, Universidad Autónoma de Nuevo León, General Escobedo 66050, Mexico; kwvazque@gmail.com

**Keywords:** ESBL, *Escherichia coli*, antimicrobial, resistance, livestock

## Abstract

**Simple Summary:**

Bacteria producing extended-spectrum β-lactamases (ESBLs) have become a critical problem of the 21st century, as they are one of the main causes of resistance to most antibiotics available on the market. This problem not only affects humans, as previously known, but has also escalated to animals, which is alarming, as many of these animals are a part of the daily diet of people worlwide. This study provides relevant information on ESBL-producing bacteria found in farm animals from different regions of the world over the last five years and may become considered an important pillar in the fight against antibiotic resistance.

**Abstract:**

β-lactam antibiotics are a key element in the treatment of bacterial infections. However, the excessive use of these antibiotics has contributed to the emergence of β-lactam-resistant enterobacteria, including *Escherichia coli*. One of the main challenges facing the public health sector is antibacterial resistance (ABR), mainly due to limited options in its pharmacological treatment. Currently, extended-spectrum β-lactamases (ESBLs) present an alarming situation, as there is an increase in morbidity and mortality rates, prolonged hospital stays, and increased costs for sanitary supplies, which involve not only humans but also the environment and animals, especially animals destined for food production. This review presents an analysis of the prevalence of ESBL-producing *E. coli* and its distribution in different animal sources throughout the world, providing an understanding of the association with resistance and virulence genes, as well as perceiving the population structure of *E. coli*.

## 1. Introduction

**Figure 1:**
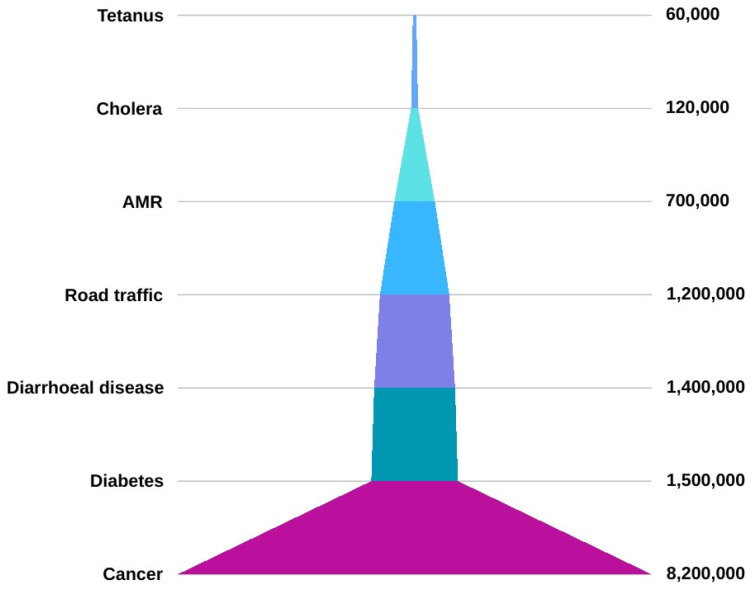
The number of deaths and their primary causes in 2019.

Antibiotics, whether produced by microorganisms or synthesized chemically, inhibit the growth and survival of bacteria [1,2]. They are crucial in modern medicine to treat infections and enable medical procedures, such as cancer treatment, organ transplants, and open-heart surgery [3]. However, most antibiotics, approximately 63,000 tons, are used in veterinary practices, with projections indicating a significant increase by 2030 [4,5]. Veterinary antibiotics, initially used for disease treatment and prevention in animals, are now commonly added to food for various purposes, including for controlling reproductive cycles and for use as prophylactics and growth promoters (GPs) [1,6,7]. Antibiotics sold without a prescription, increased international travel, and the release of unmetabolized antibiotics or their residues into the environment through manure/feces contribute to genetic selection pressure and the emergence of antimicrobial-resistant organisms [8]. Antimicrobial resistance refers to the ability of microorganisms, including bacteria, viruses, fungi, and parasites, to survive and grow despite the presence of drugs intended to kill them [9]. The World Health Organization (WHO) categorizes antimicrobial resistance as one of the top ten public health threats [10]. Deaths from antimicrobial resistance are projected to rise from 700,000 to 10 million annually by 2050, costing about USD 100 billion in healthcare resources, surpassing diseases such as cancer (Figure 1) [11,12].

Many of the antimicrobial agents used in livestock, such as third- and fourth-generation cephalosporins, fluoroquinolone, gentamicin, and erythromycin, are categorized as “critically important antimicrobials” by the World Health Organization (WHO) [13]. Under the World Organization for Animal Health (OIE) Guidelines, antimicrobials are categorized as “veterinary critically important antimicrobials”, underscoring the significance of these agents in both human and animal therapies [14]. Considering that the use of antimicrobials is the highest in poultry, followed by pigs and cattle [15,16], bacteria carrying resistant genes from animals can be transmitted to humans through the consumption of foods of animal origin. The transmission of resistant bacteria is favored by inadequate cooking of raw foods, by handling raw foods, by cross-contamination with other foods, or indirectly through the environment [17]. Nevertheless, the WHO published a list of the most critical antimicrobial-resistant microorganisms, with extended-spectrum β-lactamase (ESBL)-producing *Enterobacteriaceae* standing out among the “Highest Priority” pathogens [18]. In recent years, several studies from different countries have reported the presence of these ESBL strains in livestock [19,20,21,22,23,24]. ESBL-producing strains are resistant to most beta-lactam antibiotics, including penicillin, 1st–4th-generation cephalosporins, and monobactams, but not cephamycins or carbapenemics, and they are inhibited by β-lactamase inhibitors, such as clavulanic acid and tazobactam [20]. They often resist other antibiotics such as aminoglycosides, fluoroquinolones, and trimethoprim/sulfamethoxazole, complicating treatment [5,25,26].

ESBLs belong to class A in Ambler’s molecular classification scheme, which is based on amino acid sequence similarity [27,28]. In the Bush-Jacoby functional classification system, they are placed in group 2be. The “2b” category includes β-lactamases such as TEM-1, TEM-2, and SHV-1, while the “e” in “2be” indicates that these β-lactamases have an extended spectrum of activity [27,28,29,30] (Table 1). Furthermore, some of the most relevant enzymes are TEM (resistance to penicillins and first-generation cephalosporins), SHV (resistance to third-generation cephalosporin, monobactam, and carbapenems), and CTX-M (resistance to penicillins, narrow-spectrum cephalosporins, and cefotaxime), encoded by the *bla*_CTX-M_, *bla*_SHV_, and *bla*_TEM_ genes, respectively [23,28,31].

In the past, ESBL-producing bacteria were limited to the intra-hospital environment, but nowadays, they are isolated from food-producing animals, including cattle, pigs, sheep, and poultry. This could indicate the possible transmission and circulation of ESBL-producing *E. coli* [21,24,32,33]. The first ESBL-*E. coli* CTX-M-3 strain was detected in a dog in Japan [34], but it was not until 2003 that the first isolation of ESBL-*E. coli* CTX-M-14, SHV-12, and CMY-12 were reported in healthy broiler chickens in Spain [35]. Since then, high prevalences have been reported in different locations worldwide, including Europe [36,37], Asia [38,39], Africa [40,41], Oceania [19], and the Americas [42,43]. To better understand ABR and its global reservoirs and transmission routes, the One Health initiative is addressed. In the 1960s, the concept was recognized as “One Medicine” by the veterinary epidemiologist Calvin Schwabe. However, the term “One Health” was first used in 2003–2004 by the Wildlife Conservation Society in response to the emergence of severe acute respiratory syndrome (SARS) and H5N1 avian influenza [44]. This approach recognizes the interconnection between human and animal health and the ecosystem in which they coexist [45].

It is imperative to maintain updated data on ESBL-*E. coli* in animals for human consumption. This information is crucial to implement new strategies for mitigation and reduction of the emergence of these bacteria. In this sense, this study focused on a comprehensive review of the impact of ESBL-*E. coli* in farm animals, as well as on analyzing possible transmission routes. Finally, solid information and knowledge are provided on the spread of this important group of bacteria, together with ESBL genes, considering the past six years across different global regions.

## 2. Extended-Spectrum β-Lactamase-Producing *E. coli*

One of the main mechanisms of bacterial resistance to β-lactam antibiotics is the production and neutralization of enzymes, particularly β-lactamases [2,46]. These enzymes were first described in England in 1940; at that time, it was noticed that *Bacillus coli* (now *E. coli*) was not inhibited by penicillin [34]. Later, it was confirmed to be the chromosomal AmpC class C cephalosporinase, according to the Ambler molecular scheme [47]. In the early 1980s, the problem worsened with the emergence of strains that produce enzymes capable of hydrolyzing third-generation cephalosporins, known as ESBLs [34]. These enzymes emerged from point mutations in the amino acid sequences of β-lactamase type SHV-1 (variable sulfhydryl, active site), TEM-1, or TEM-2 (isolated from clinical samples at Temoneira in Athens, Greece), leading to a broader spectrum of activity, especially to penicillins and oxymino-cephalosporins [34,48,49].

In recent decades, ESBL-*E. coli* has been isolated from food-producing animals in in-hospital and community settings [5,40,50,51,52]. This has caused great concern, as these animals can act as reservoirs and vehicles for the transmission and dissemination of these bacteria. The first isolation of an ESBL strain in animals occurred in Japan in 1988, with a CTX-M-3-producing *E. coli* strain isolated from a dog, marking the beginning of its global spread [34,53]. For example, in Europe, from 2000 onwards, the European Antimicrobial Resistance Surveillance Network reported an increase in the rates of oxymino-cephalosporin-resistant *E. coli* and *Klebsiella pneumoniae* isolates [54]. This development was also noticeable in the animal sector, mainly in swine production in Europe [6]. Additionally, this industry may contribute to the spread of antibiotic-resistant bacteria. In industrialized countries, intensive pig farming is common, leading to cramped conditions, limited mobility, and the risk of mixing with sick pigs [4,6]. Moreover, since 2008, ESBL-producing bacteria have been recognized as a key factor in mastitis among dairy cows [55]. With these data, we can observe that ESBL-*E. coli* strains are distributed globally, showing enzymatic diversity and genetic variability. Their presence in animal samples signals a serious issue, especially considering that these animals are destined for food production. This suggests that epidemiological surveillance efforts are crucial due to the magnitude of the problem.

## 3. Publications Analyzed

For the scientific literature search, PubMed, ScienceDirect, and Google Scholar were used to find articles published in English from January 1, 2018, to October 31, 2023. The search terms included the following: “*Escherichia coli*”, “*E. coli*”, “extended-spectrum beta-lactamase-producing *E. coli* in animals”, “ESBL-producing *E. coli* in livestock”, “extended-spectrum beta-lactamase-producing *E. coli* in cows”, “extended-spectrum beta-lactamase-producing *E. coli* in poultry”, “extended-spectrum beta-lactamase-producing *E. coli* in pigs”, and “extended-spectrum beta-lactamase-producing *E. coli* in sheep”. Only research articles and reviews were included, excluding letters to the editor, conference abstracts, and book chapters. The exclusion criteria were (1) papers that did not clearly show information on the sample and ESBL-*E. coli* isolate numbers. The inclusion criteria were (2) studies focusing on ESBL-*E. coli* in farm animals (cows, chickens, pigs, and sheep), some of which including human samples, and (3) studies using phenotypic or molecular tests for the confirmation of ESBL. Data were extracted and compiled in a Microsoft Excel spreadsheet, including the author’s last name, publication year, sample type and number, detection method (phenotypic/genotypic), ESBL-*E. coli* prevalence, antibiotic resistance, and percentage of ESBL-encoding genes. 

**Figure 2:**
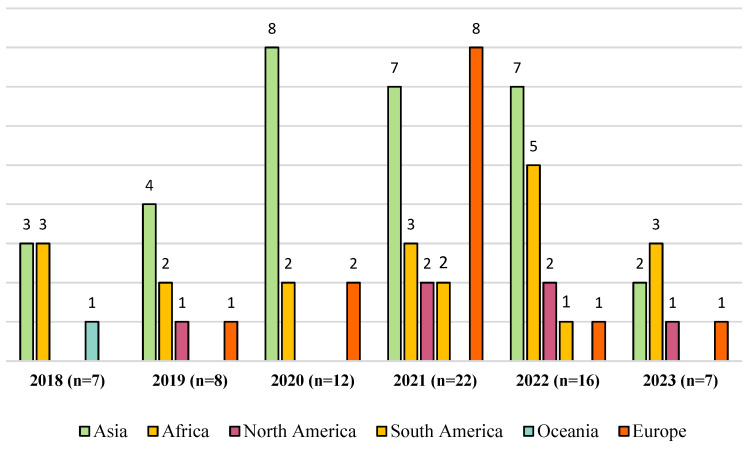
Classification of the 72 studies included in this review, which were found in PubMed, ScienceDirect, Google Scholar, and additional databases from 2018 to 2023. The colored bars represent the number of studies conducted in different geographic regions around the world.

Seventy-two scientific studies were grouped by year of publication and the source of the sample (Figure 2). In 2018, most studies focused on pigs (57.1%; 4/7) and were mainly conducted in Asian countries [40,56,57,58]. Only one work was conducted in Oceania [19]. In 2019, studies were also mainly conducted in Asian countries (50.0%; 4/8); however, they focused more on poultry [31,59,60,61]. In 2020, Asia had the highest number of studies focusing on ESBL-*E. coli* strains isolated from three high-demand farm animals, namely, chickens, cows, and pigs (66.7%; 8/12) [38,39,62,63,64,65,66,67], surpassing countries in Europe and the Americas. In 2021, a higher number of studies related to pigs, chickens, cows, and sheep were obtained, where Europe led in quantity (36.4%; 8/22) [4,21,68,69,70,71,72,73] compared to Asia, Africa, and America. One year later, the number of articles decreased, but a bias towards Asian countries was observed (43.8% 7/16) [33,74,75,76,77,78,79]. In 2023, only seven articles were found, mainly in Africa (42.9%; 3/7) [50,80,81].

## 4. Antibiotic Resistance in Livestock

In bovines, high percentages (>60.0%) of resistance to ampicillin (AMP), cefepime (FEP), cefotaxime (CTX), tetracycline (TET), and gentamicin (GEN) have been reported in several countries across five continents [22,36,39,43,56,68,73,77,82,83,84].

Within the pork industry, several countries in Asia, Africa, and the Americas have reported high percentages of resistance to AMP, CTX, and ceftazidime (CAZ) [40,59]. Additionally, resistance rates exceeding 70.0% have been reported for TET, amoxicillin (AMX), erythromycin (ERY), and GEN [22,58,64,75,85]. In Europe, nations such as Spain, France, the Netherlands, Hungary, Italy, and Latvia display elevated rates (≥90.0%) of resistance to β-lactam antibiotics, such as AMP and CTX [4,21,69,73], as well as resistance to non-β-lactam antibiotics, including TET, exceeding 60.0% [71].

In sheep, although rare worldwide, studies have reported high resistance ratios (>70.0%) to TET, GEN, streptomycin (STR), trimethoprim–sulfamethoxazole (SXT), and CAZ [36,50,70,79,86].

The poultry industry has shown high resistance percentages in Asia, with more than 90.0% of isolated strains showing phenotypic resistance to the non-β-lactam antibiotics AMP, CTX, and CAZ [39,61] and over 80.0% showing phenotypic resistance to the non-β-lactam antibiotics TET, SXT, GEN, and nalidixic acid (NAL) [59,60,87,88]. In African countries, high rates of phenotypic resistance are reported in broiler chickens [40,50,52,89]. In North and South America, poultry studies have reported resistance percentages higher than 80.0% for CTX, TET, GEN, and STR [86,90,91]. Baez et al. suggested that the high antibiotic resistance in undeveloped countries is due to the use of antibiotics to treat respiratory diseases and intestinal infections despite their prohibition in some countries [90].

The rise of multidrug-resistant (MDR) bacteria poses a global challenge linked to poor antibiotic management, particularly in food animals [8]. In this regard, high percentages of antibiotic resistance have been observed among *E. coli* strains, notably in pigs and poultry [73,86]. Results indicate that the swine, poultry, and cattle industries exhibit the highest rates of antibiotic resistance. This is supported by authors such as Bergšpica et al. [6], who note that the slaughter process involves several stages that can increase the risk of contamination. In contrast, lower percentages of antibiotic resistance are observed in sheep, a sector that is not as frequently studied [36,70]. Therefore, rational antibiotic use is crucial in the livestock industry.

## 5. Prevalence of ESBL-*E. coli* in Livestock

Africa reported the highest number of countries with ESBL-*E. coli* isolated from cattle between 2018 and 2023. In Ivory Coast in 2018, the highest prevalence (52.9%) was recorded [82], while in Tunisia in 2019, the lowest prevalence (16.1%) was reported [50]. In Asia, ESBL-*E. coli* prevalence reached 66.4% in 2022 in countries such as China and Pakistan [75,77], with the lowest figure being 3.7% in 2020 in Malaysia and South Korea [39,62]. In the Americas, in the northern region, such as the United States, a higher prevalence of 48.3% was observed [92], in contrast to the southern region, where a prevalence of 3.0% was reported in countries such as Chile [20]. In Spain, France, the United Kingdom, the Netherlands, and Italy, ESBL-*E. coli* was described in cattle, with the highest value (24.5%) found in 2023 [93].

Pig production is widespread throughout the world, especially in industrialized countries, where these animals are raised on farms that provide controlled conditions [4,6]. In a six-year retrospective, Africa reported the highest prevalence (65.3%) of ESBL-*E. coli* in pigs in 2021 in Tanzania [85]. However, in Asia, the highest figure (98.0%) was reported in 2019 in Thailand [59]. In the Americas, few studies focusing on ESBL-*E. coli* in pigs have been conducted, especially in the northern region, where countries such as the United States [22] and Mexico reported a similar prevalence of approximately 7.0% [86]. In Oceania, specifically in the Australasian subregion, few reports of ESBL-*E. coli* in farm animals, especially pigs, are expected. However, Abraham et al. reported a 35.9% prevalence rate for Australia [19]. In Europe in 2021, papers focused on pigs were observed in Greece, France, Germany, Spain, the United Kingdom, Italy, and Hungary, with an average prevalence of 18.8% for ESBL-*E. coli* isolated from swine fecal samples [69,71,73]. Some of these studies also involved humans [59].

Sheep farming is presumed to be important for feed and textile production in the agricultural sector. However, antimicrobial resistance affects this sector around the world [70]. Studies on the prevalence of ESBL-*E. coli* isolated from sheep are limited, as noted by Tello et al. [36] and Dantas Palmeira et al. [70]. Africa, notably Nigeria, has driven much of this research. For example, Olorunleke et al. reported a prevalence of up to 56.0% for ESBL-*E. coli*, which is among the highest recorded (2018–2023) [84]. China [79] and Pakistan [77] averaged 24.4%. In the Americas, Mexico [86] and Chile reported similar (~3.0%) rates [20]. In Europe in 2021, the highest ESBL-*E. coli* prevalence records were observed in sheep on farms in southern Portugal (90.5%) [70].

**Figure 3:**
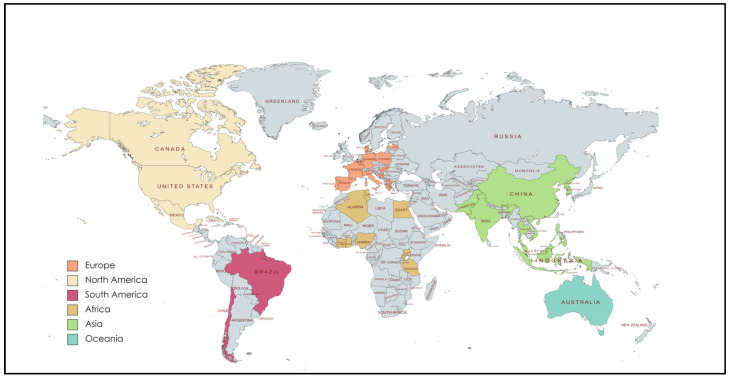
World map showing the countries with studies on ESBL-*E. coli* in livestock.

**Figure 4:**
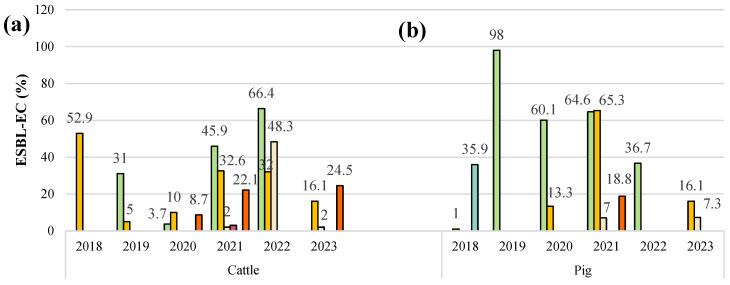

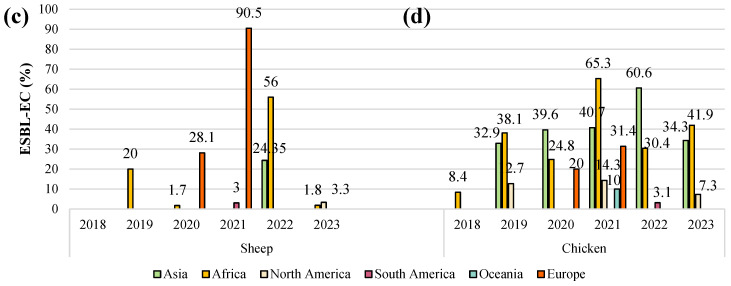
Prevalence of ESBL-*E. coli* isolates according to their sample source: (**a**) cattle, (**b**) pig, (**c**) sheep, and (**d**) chicken. Contrasting prevalence of ESBL-*E. coli* by phenotypic and genotypic methods in 72 studies worldwide during the period from 2018 to 2023.

The poultry industry is affected by the emergence of ESBL-producing bacteria. Africa, including Nigeria [51], Egypt [24], and Tanzania [85], has reported ESBL-*E. coli* presence in chickens. The highest record (65.3%) was found in 2021 in Tanzania [85]. Asia has numerous reports of ESBL-*E. coli* isolated from poultry [76], with high prevalence rates in India, Pakistan, Malaysia, and Indonesia, averaging 60.6% [33,61,75,94]. In the Americas, countries such as Chile [20], the United States [22], and Cuba [90] have noted ESBL-*E. coli* in broiler chickens, with an average prevalence of 14.3%. Europe observed its highest ESBL-*E. coli* prevalence in chickens in 2021, with countries such as Hungary, Belgium, Denmark, France, Germany, Bosnia, Spain, Poland, Italy, and the United Kingdom showing an average prevalence of 31.4% [4,21,69,71,72,73]. The variation in the prevalences of ESBL-*E. coli* isolated from farm animals worldwide could be attributed to factors such as climate, customs, traditions, animal management, and antibiotic use (Figure 3 and Figure 4).

## 6. Distribution of Genetically Variant ESBLs

Approximately 4900 β-lactamases have been reported as unique enzymes, with the number of variants growing exponentially due to whole-genome sequencing (WGS) studies [30]. Several genes encoding ESBLs, such as *bla*_TEM_, *bla*_SHV_, *bla*_CTX-M_, *bla*_GES_, *bla*_VEB_, *bla*_IRT_, *bla*_CMT_, *bla*_BEL_, *bla*_TLA_, and *bla*_PER_, have garnered attention due to their current public health impact [95]. However, the most relevant gene families detected in animals are TEM, SHV, and CTX-M [29,95]. TEM- (243 variants) and SHV-type ESBLs (228 variants) are closely related, differing only in a single amino acid substitution [30]. In the early 2000s, CTX-M-type enzymes diversified worldwide, displacing TEM and SHV and becoming the most prevalent ESBL type in some *Enterobacterales* members, possibly originating from chromosomal β-lactamases of several species of the genus *Kluyvera* [49,96]. To date, CTX-M-type enzymes include approximately 230 variants subdivided into five groups according to amino acid sequence homology: CTX-M-1, CTX-M-2, CTX-M-8, CTX-M-9, and CTX-M-25 [20,29,30]. ESBL-encoding genes have been identified in several geographical locations in both humans and animals by PCR or WGS [19,59,61,92]. For example, major regions such as the United States, Brazil, the European Union, China, and India reported beef cattle carrying commensal or clinical ESBL-*E. coli* [55]. In Asia, CTX-M-1, CTX-M-15, and CTX-M-9 were described in cows from South Korea and Pakistan, while the TEM gene was detected in Pakistan, Malaysia, and China [39,60,77]. Not surprisingly, the CTX-M-15 gene was detected in South Korea, and this gene and CTX-M-14 are the most abundant CTX-M-type β-lactamases among the ESBL-*E. coli* strains isolated from livestock [39,97]. The emergence of the CTX-M-15 gene has caused controversy worldwide and is associated with multidrug-resistant (MDR) strains in both community and hospital settings [60].

In Africa, the CTX-M genes, specifically the CTX-M-14 and CTX-M-15 variants, were prevalently detected in Nigeria [83,84,98]. CTX-M-15 is the dominant gene among ESBL-*E. coli* strains isolated from humans [84,99], and it is frequently observed in farm animals worldwide. It is noted that this gene is more diversified across locations and sources than other CTX-M types [98]. Meanwhile, in the Americas, the first isolates of ESBL-*E. coli* carrying CTX-M emerged in cows in the United States in 2010. Today, CTX-M-1 and CTX-M-9 variants are recorded in Mexico and the United States. The TEM gene is found in smaller proportions. The SHV gene was identified in only one study in Chile [20,22,86,92]. As expected, the CTX-M family, mainly CTX-M-1 and CTX-M-15, predominates in Spain, France, Germany, the Netherlands, Italy, and Macedonia, followed by TEM (including TEM-1 and TEM-52), with the SHV and OXA genes in smaller proportions [21,36,71,73,93]. The CTX-M-1 and CTX-M-15 genes were the most common ESBL types in cattle in a study conducted by Giufrè et al. [21]; these enzymes were detected in both animals and humans, suggesting the horizontal transfer of ESBL genes between these sources [21].

In the Asian swine industry, the CTX-M-14, CTX-M-15, and CTX-M-55 genes were detected mainly in China and Thailand, and the TEM, SHV, and OXA genes were also found [57,59,75,78,100,101]. Hammerum et al. previously reported the emergence of these genes in Asian countries [102]. In Africa, only Nigeria and Tanzania reported the CTX-M gene, while the TEM and SHV genes were not detected [40,85]. In North and South America, few ESBL gene descriptions were reported, especially the CTX-M type [20,86]. Only one paper in Chile and one in Mexico reported ESBL-*E. coli* strains harboring the TEM and SHV genes [20,22,86]. In Oceania, only Australia reported the CTX-M-1 gene in 100.0.% of ESBL-*E. coli* strains [19]. However, European countries such as Greece, Hungary, France, Germany, and the Netherlands recorded high proportions of the CTX-M-1 and CTX-M-15 genes, the most prevalent ESBL genes among farm animals in Europe [69,71,73,103]. The TEM gene followed, with only Greece and Latvia reporting the SHV gene [4,69]. Previous research by von Salviati et al. [104] also documented the presence of these genes in German pig farms.

In eastern sheep, China and Pakistan reported CTX-M-1, CTX-M-55, CTX-M-15, CTX-M-9, TEM-1, OXA-1, and SHV [70,77]. In Africa, only two countries reported the CTX-M-1 and CTX-M-15 genes, along with the TEM-1 gene [50,84]. In the Americas, Mexico described ESBL-*E. coli* in sheep, detecting only CTX-M, with no presence of TEM or SHV [86]. In Europe, countries such as Portugal and Spain detected CTX-M variants such as CTX-M-14, CTX-M-15, and CTX-M-32, as well as TEM, SHV-12, and OXA-1 [36,37]. The coexistence of multiple CTX-M β-lactamase types in the same strain is common due to shared multiple homologous regions that could give rise to the emergence of recombinant genes [31].

In the Asian poultry industry, ESBL-*E. coli* strains were found to mostly harbor the CTX-M gene, including the variants CTX-M-1, CTX-M-14, and CTX-M-15 [31,60,61,63]. Furthermore, the CTX-M-55 gene was observed in Thailand, Pakistan, and China [60,61,63]. In this sense, the epidemiology of CTX-M-type β-lactamases has evolved, with an example of this being the CTX-M-55 gene, differing by a single nucleotide at position 239, obtaining A77V, reflecting higher hydrolyzing activity against some cephalosporins [38,105]. Several countries reported elevated TEM gene numbers, with these genes being predominant in China [38,39,87,94].

In Africa, the presence of CTX-M genes, mostly CTX-M-15, was described as expected due to their wide diversification in humans and animal species (including poultry) globally, including the African continent [40,80,83,106]. Only in several studies in Egypt were the TEM, SHV, and OXA genes identified [5,24,52].

In North and South America, more poultry-related studies were noted, including in Canada, the United States, Cuba, and Brazil. CTX-M-15 and CTX-M-1 were described as prevalent genes, with CTX-M-1 being the most common worldwide in ESBL-*E. coli* strains isolated from chickens [22,86,90,107]. Different genes were reported in South America, including CTX-M-2, CTX-M-8, CTX-M-65, CTX-M-55, and CTX-M-3 [22,86,90,107,108]. The TEM gene was reported in the United States, Mexico, and Chile, and the SHV gene was only detected in Brazil and Chile, which was surprising because of the high occurrence of CTX-M [20,91]. In Europe, most of the ESBL-*E. coli* strains isolated from chickens harbored the CTX-M group 1 and CTX-M-15 genes, with countries such as France, Spain, Italy, and the Netherlands reporting the presence of the TEM gene but not detecting the SHV gene [37,69,71,72,73]. Moreover, Alegría et al. detected the CTX-M-14 gene in both clinical and food samples in Spain [37]. More details of these results can be found in Table 2.

These results provide precise information on the widespread distribution of ESBL gene types in food-producing animals worldwide. Most authors detected CTX-M genes or their variants, primarily in different Asian regions, highlighting high diversity across animal sources. This was followed by the TEM gene and its variants, with few studies identifying the SHV gene. The diversity and abundance of these resistance genes highlight the complexity of the issue, emphasizing the need for comprehensive control and surveillance strategies to minimize their spread and impact on human and animal health.

## 7. Circulation of Non-β-Lactam Resistance Genes

In addition, ESBL genes are carried on large plasmids, which can also harbor other genes resistant to various groups of antibiotics, including tetracyclines, fluoroquinolones, aminoglycosides, and sulfonamides [20,114]. These mobile genetic elements (MGEs) can transfer genes between bacterial species and hosts, such as humans and animals, limiting treatment options [85]. Studies worldwide have demonstrated the existence of these genetic determinants in animals destined for food production [52,88,92,93]. For instance, in the Asian bovine industry, countries such as Pakistan have seen an increase in resistance to colistin, originated by the *mcr*-1 (37.8%) gene [77]. This antibiotic is considered a last resort in the treatment of infections caused by MDR bacteria; however, deliberate use in animals is leading to resistance mechanisms [77].

In Africa, ESBL-*E. coli* strains isolated from cows were reported to harbor several antibiotic resistance genes (ARGs), including *str*B (>80.0%), *sul*2 (>70.0%), and *qnr*S1 (>77.0%), detected using PCR and WGS [84,85,99]. The latter gene was detected in Nigeria at high percentages, and it confers resistance to quinolones [83]. Aworh et al. and Olorunleke et al. [84] suggest this may result from excessive antibiotic use, mainly for prophylactic purposes in livestock production in Nigeria, considering the decrease in fluoroquinolone use in Europe [83,84].

The *tet*A (>70.0%), *str*B (100.0%), *qnr*B (>40.0%), and *aad*A1 (60.0%) genes were identified in ESBL-*E. coli* strains in bovines, mainly in North American countries such as the United States and Mexico [22,86,92]. The high prevalence of the *tet*A gene was anticipated due to the widespread use of tetracycline, one of the most affordable antibiotics, in human and animal infections [22]. In European countries such as Spain, Greece, France, Germany, and the Netherlands, some ARGs coexist with ESBL genes, most frequently *tet*A (>70.0%), *sul*2 (>80.0%), and *aad*A1 (>40.0%) [36,68,71,73].

In the Asian swine industry, the genes *qnr*S1 (>67.0%), *aad*A1 (>65.0%), *sul*1, *sul*2, *sul*3 (>50.0%), *aph* (3’)-Ia, *aph* (6’)-Id (>48.0%), *qnr*A (>34.0%), and *tet*A (22.0%) were described [57,59,64,101]. Furthermore, the coexistence of *qnr* and *bla*CTX-M genes on the same plasmid has been reported, potentially contributing to the diversification of qnr genes in Asian countries [59,115]. Vietnam and Thailand reported the presence of the *mcr*-1 (>41.0%) gene in ESBL-*E. coli* strains isolated from pigs [100,101]. High prevalences of the *qnr*S1 (82.0%), *sul*2 (75.0%), and *qnr*B (10.0%) genes were observed in Africa [84,85]. The high prevalence of *qnr*S1 in African countries such as Nigeria may be attributed to the wide application of fluoroquinolones, particularly in the absence of antibiotic regulations for pigs, which could potentially contribute to AMR in *E. coli*, whereas in Europe, where antibiotic use has significantly decreased, the prevalence of ESBL-*E. coli* is lower [84]. In the Americas, particularly in Northern countries such as the United States and Mexico, high prevalences were observed in the *tet*A (73.0%), *aad*A1 (60.0%), *sul*2 (42.9%), and *aph*(6)-Id (28.6%) genes [22,86,90]. In Oceania, a study in Australia recorded high percentages for *aad*A5, *dfrA17* (100.0%), and *sul*2 (98.3%) genes [19]. This study also indicated the *flo*R (4.9%) gene in a few ESBL-*E. coli* strains, which confers resistance to florfenciol; it is worth noting that this antibiotic is not administered to humans but is used to treat various infections in animals [19]. In Europe, high prevalences were reported for the *tet*A (>27.0%), *sul*1 (57.1%), *sul*2 (44.4%), *dfr*A5 (63.6%), and *aad*A1(>43.0%) genes [68,71,73,103]. In their study on a Danish pig farm, Jensen et al. [116] showed that the use of tetracycline, although not critically important in human medicine, could eventually lead to antibiotic resistance, posing a threat to the health sector [116].

Studies on ARGs detected in ESBL-*E. coli* strains isolated from sheep worldwide are very rare. For example, Asian countries such as Pakistan and China noted the appearance of ARGs such as *aph*(6)-Id (45.0%), *tet*A (42.0%), and *sul*2 (43.2%), and a small proportion of ESBL-*E. coli* strains had the *mcr*-1 (7.4%) gene [77,79]. In Africa, specifically in Nigeria, several ARGs were identified using WGS, including *str*B (70.0%), *sul*2 (70.0%), and *qnr*S1 (60.0%); antibiotics such as aminoglycosides and sulfamethoxazole are widely applied in farm animals [84]. In America, *tet*A (73.3%) and *aad*A1 (60.0%) genes showed a higher prevalence [86]. In Europe, Spain and Portugal documented some ARGs, including *tet*A (>51.0%), *tet*B (29.1%), *sul*1 (42.9%), *sul*2 (81.6%), *sul*3 (24.5%), and *aac*(6’)Ib-cr [36,70]. The appearance of the *sul*3 gene is surprising because it is very rare in animals [79].

In the poultry industry in Asia, there are numerous reports on the high prevalence rates of *tet*A (>40.0%), *sul*2 (>75.0%), *qnr*S, *qnr*A, *aac*(6’*)*-Ib-cr, *dfr*A14, and *mcr*-1 (<35.0%) genes, identified using PCR or WGS [76,77,88,112]. Notably, a high percentage of acquired sulfonamide resistance has been observed, with some in silico studies offering valuable information on the *E. coli* resistome isolated from fecal samples [76]. Additionally, Lemlem et al. [88] reported the coexistence of the *mcr*-1 gene and *bla*_CTX-M_ in the same plasmid. In African countries, ESBL-*E. coli* isolated from chickens exhibited a diverse array of ARGs, including *tet*A (>55.0%), *tet*B (>55.0%), *sul*1 (62.0%), *sul*2 (>66.0%), *aad*A1(80.0%), *mdf*A (macrolide resistance) (>91.0%), *aac*(6’)-Ib-cr (aminoglycoside and quinolone resistance) (>15.0%), *aph*(6)-Id (>59.0%), and *flo*R (florfenicol resistance) (>66.0%) [40,80,85,89,109]. These high percentages may be due to the fact that these classes of antibiotics are commonly used in poultry farms for therapeutic purposes in Nigeria. Additionally, some authors have noted the spread of these genes between strains facilitated by MGEs such as plasmids or integrons [85,89]. In America, few reports exist, but *tet*A (>50.0%), *tet*B (>40.0%), *sul*2 (>37.0%), *aad*A1 (60.0%), and *mph*(A) (50.0%) genes has been detected [86,90]. Europe shows a higher prevalence of *tet*A (52.5%), *sul*2 (44.4%), and *aad*A1 (43.4%) genes [71,73], as outlined in Table 2. The presence of ESBL-*E. coli* isolated from farm animals, with various ARGs, has been reported in several countries worldwide [5,22,37,79]. An analysis of the studies included in this review indicates a notable prevalence of genes associated with resistance to tetracyclines, sulfonamides, and streptomycin in the farm animals studied, with a higher frequency observed in studies conducted in African and Asian regions. This could be attributed to the irrational use of antibiotics for growth promotion and the prevention and treatment of animal diseases [6,14]. The diversity and abundance of these resistance genes emphasize the complexity of the problem, stressing the need for continued epidemiological surveillance strategies to monitor these ARGs in ESBL-*E. coli*.

## 8. Virulence Factors

*E. coli* has several virulence factors (VFs) encoded by chromosomal genes or located in MGEs, leading to various intestinal and extraintestinal disorders in humans and animals globally [117]. These factors are categorized into two subdivisions. The first focuses on pathogenic *E. coli* causing gastrointestinal diseases, comprising seven pathotypes: enteropathogenic *E. coli* (EPEC), which causes diarrheal diseases in infants; enterotoxigenic *E. coli* (ETEC), which is described to release enterotoxins in the intestine and is associated with traveler’s diarrhea and diarrhea in children in developing countries; enterohemorrhagic *E. coli* (EHEC), which is associated with food-borne outbreaks and causing hemorrhagic colitis (HC) and hemolytic uremic syndrome (HUS); Shiga-toxigenic *E. coli* (STEC), which produces Shiga toxins and can lead to HUS; diffusely adherent *E. coli* (DAEC), which is associated with urinary tract infection (UTI) in adults, diarrhea in children, and complications in pregnant women; enteroaggregative *E. coli* (EAEC), which is food-borne and associated with acute diarrheal infections in children and immunocompromised individuals; and entero-invasive *E. coli* (EIEC), which is food-borne and causes mucous and bloody diarrhea and invasive inflammatory colitis [117,118]. The second subdivision comprises avian pathogenic *E. coli* (APEC), which is associated with respiratory infections and septicemia in poultry and is responsible for significant morbidity and mortality in the poultry industry worldwide, and uropathogenic *E. coli* (UPEC), which is responsible for UTIs in humans [117,118].

In Asian countries such as Pakistan, three pathotypes have been identified among ESBL-*E. coli* strains isolated from cattle: EHEC, with the highest number of strains, followed by EPEC and EAEC [77]. STEC infections in humans are almost always caused by direct or indirect contact with food or water contaminated with cattle feces [77]. In Africa, Fashae et al. [98] reported that ESBL-*E. coli* strains in Nigerian cattle showed the presence of several VFs, such as Glutamate-1-semialdehyde (*hem*L), followed by increased serum survival (*iss*), and less than 30% had the probable major fimbrial (*lpf*A), a potential virulence marker in *E. coli* aiding in the formation of microcolonies in all strains [98]. The α-hemolysin (*hlyA*), *fim*, *csg*, and flagellar basal-body rod protein (*flg*) were described in ESBL-*E. coli* isolated from cows in North America, including the United States and Mexico [22,42,43]. Some are also reported in Europe and Africa. *E. coli hlyA* is clinically relevant, as it produces a toxin capable of lysing erythrocytes and leukocytes, and there is an increased proliferation of these bacteria in active ulcerative colitis in the mucosa of the human colon [119]. European cattle exhibited several in silico VFs identified in more than 80% of ESBL-*E. coli* strains in countries such as Spain, Belgium, Denmark, France, Germany, Hungary, the Netherlands, and the United Kingdom, including enterobactin siderophore (*ent* A, B, C, D, E, F, G, H, and I), type I fimbriae (*fim* A, B, C, D, E, F, G, H, and I), curli fiber (*csg* A, B, C, D, E, F, and S), and, in smaller numbers, the manganese ABC transporter system (*sit* A) [71]. The high prevalence of the *fim* gene is important due to its relevance to the pathogenesis of extraintestinal diseases of *E. coli*, particularly biofilm formation [119].

The VFs *tra*T, *lpf*A outer membrane protease (*omp*T), Fe, Mn transporter (*sitA*), and *iss* were described in ESBL-*E. coli* in Asian pigs [101]. In the Americas, Ibekwe et al. described some VFs in swine in the United States using WGS, including *flg*, *fim*, and *csg*, highlighting that one strain harbored more than 100 VFs [22]. Conversely, in Mexico, Mandujano et al. reported only the presence of the *hly*A gene and the absence of the *stx*1 and *stx*2 genes among ESBL-*E. coli* strains, marking one of the first studies in farm animals in this country [86]. In Oceania, Abraham et al. studied pigs randomly selected from an Australian piggery between 2013 and 2016 [19]. They found that most ESBL-*E. coli* strains carried the *eae* gene, which encodes intimin associated with intestinal colonization and increases the risk of developing HUS. The detection of the *eae* gene or *stx*1 and *stx*2 could indicate strains with high pathogenic potential [120].

Studies analyzing different VFs in ESBL-*E. coli* strains isolated from sheep are uncommon, and no reports were found in Europe or Africa. In Asia, only the following pathotypes are described by their prevalence percentage: EHEC with the highest number of strains, followed by EPEC and EAEC [77]. In the Americas, Mandujano et al. conducted a study in Mexico focused on farm animals, including sheep, finding that >40.0% of ESBL-*E. coli* strains harbored the *hly*A gene [86].

In the poultry industry in Europe, Ewers et al. reported ESBL-*E. coli* in multiple countries [71]. They used WGS and found that >90.0% carried various VFs, including genes encoding the enterobactin siderophore (*ent* A, B, C, D, E, F, G, H, and I); type I fimbriae (*fim* A, B, C, D, E, F, G, H, and I); and curli fiber (*csg* A, B, C, D, E, F, and S), many of which are associated with APEC, representing a threat to public health [71]. In Africa, limited research addressed VF profiles in ESBL-*E. coli* strains isolated from chickens. One such study, by Benlabidi et al. [80], investigated ESBL-*E. coli* occurrence in poultry from a rural area in Tunisia. They detected some genes encoding VFs commonly found in pathogenic *E. coli*. Over 70.0% of the strains exhibited VFs such as *fim*H, *fyu*A encoding the yersiniabactin receptor, and *iut*A encoding the ferric aerobactin receptor. The authors suggest that there is a diversity of genetic determinants, including VFs, among ESBL-*E. coli* strains, reflecting significant genetic dynamics in the bacterial population [80]. In Asia, various VFs have been reported, including colicin-lb (*cib*), a transmembrane toxin involved in the depolarization of the cytoplasmic membrane; tellurium ion resistance (*ter*C); and transfer protein (*tra*T), which inhibits the classical pathway of complement activity [76].

Furthermore, in a study by Shafiq et al. on Pakistani chickens, all ESBL-*E. coli* strains were tested for six VFs, finding that more than 40.0% of the strains were identified as EHEC [77]. In the Americas, only the *hly*A gene was reported [86]. Conversely, VFs—including *ent* A, B, C, D, E, F, F, G, H, and I; *fim* A, B, C, D, E, F, G, H, and I; *csg* A, B, C, D, E, F, and S; and *fim* A, B, C, D, E, F, G, H, and I—were also detected in over 90% of the ESBL-*E. coli* strains isolated from pigs in the swine industry across European countries, including Belgium, Denmark, France, Germany, Hungary, Poland, the Netherlands, Spain, and the United Kingdom [71]. These results are detailed in Table 2.

Importantly, recent studies on the VFs in ESBL-*E. coli* isolated from farm animals globally are limited. However, these results suggest that the diversity of VFs in ESBL-*E. coli* may result from adaptive strategies developed in response to hostile environments, particularly the selective pressure exerted by antibiotics. These findings raise concerns regarding food safety and public health. Therefore, it is necessary to continue to investigate and monitor the presence of these VFs in ESBL-*E. coli* in order to understand their impact on animal and human health.

## 9. Conclusions

To the best of our knowledge, ESBL-producing bacteria isolated from hospital settings have been extensively studied. However, this review confirms that farm animals worldwide can serve as reservoirs for ESBL-*E. coli*. Common sources include chickens and pigs, especially in regions such as Asia and Africa. The prevalence of ESBL-*E. coli* varies significantly between regions, influenced by factors such as climate, antibiotic usage, and animal husbandry practices. Moreover, ESBL-*E. coli* strains harbor various genetic determinants, including ESBL genes (TEM, CTX-M, and SHV), non-ESBL antibiotic resistance genes, and virulence-associated genes, enhancing colonization within hosts. The detection of ESBL-*E. coli* in farm animals emphasizes the need for national surveillance programs focused on epidemiological monitoring, particularly in developing countries, to track the spread of MDR bacteria. Promoting multisectoral and multidisciplinary cooperation is crucial to effectively address this global health challenge.

## Figures and Tables

**Table 1:** Classification schemes for bacterial β-lactamases (adapted from Tang [28]; De Angelis [30]).

Ambler Scheme (Molecular Class)	Bush–Jacoby Group (Functional)	Characteristics	Substrate	Inhibited by		Enzyme Examples
				Clavulanic Acid	EDTA	
A	2a	Penicillinases	Penicillins	+	-	PC1
	2b	Broad-spectrum enzymes	Penicillins, early cephalosporins	+	-	TEM-1, TEM-2, TEM-13, SHV-1, SHV-11
	2be	Extended broad-spectrum enzymes	Penicillins, oxyimino-cephalosporins, monobactams	+	-	TEM-3, TEM-10, SHV-2, CTX-M, PER-1, VEB-1
	2br	Broad-spectrum enzymes	Penicillins	-	-	TEM-30, TEM-31, SHV-10
	2ber	Extended-spectrum enzymes	Penicillins, extended-spectrum cephalosporins, monobactams	-	-	TEM-50, TEM-158
	2c	Carbenicillin-hydrolyzing enzymes	Penicillins, carbenicillin	+	-	PSE-1, CARB-3
	2ce	Extended-spectrum carbenicillinase	Carbenicillin, cefepime	+	-	CARB-10
	2e	Cephalosporinases	Extended-spectrum cephalosporins	+	-	CepA
	2f	Carbapenem-hydrolyzing nonmetallo-β-lactamases	Carbapenems	+	-	GES, KPC-2, SME-1, IMI-1
B	3	Metallo-β-lactamases	Carbapenems	-	+	IMP, VIM, IND
C	1	Cephalosporinases	Narrow- and extended-spectrum cephalosporins	-	-	MIR-1, ACT-1, FOX-1, CMY-47
D	2d	Cloxacillin-hydrolyzing enzymes	Cloxacilina, oxacilina	+	-	OXA-1, OXA-10
	2de		Cloxacillin, oxacillin, oxyimino-cephalosporins, monobactams	+	-	OXA-11, OXA-15
	2df		Carbapenems	+	-	OXA-23, OXA-51, OXA-58

**Table 2:** Molecular characteristics of ESBL-*E. coli* isolated from livestock between 2018 and 2023.

Continent	Country	Source	Detection Test	ESBL Genes	Other Antibiotic Resistance Genes	Virulence Genes	Reference
Africa	Nigeria	Poultry	PCR	CTX-M-1, CTX-M-55, TEM	*tet*A, *tet*B, *aac(3)-II*	-	[40]
		Pig	PCR	CTX-M-15	-	-	
	Egypt	Poultry	PCR, microarrays	TEM, SHV, OXA-1, CTX-M-1, CTX-M-15	*aadA*1, *sul*2, *flo*R, *qnr*S, *qnr*B, *dfr*A, *sul*3, *tet*A, *tet*B, *tet*C	*lpf*A, *hem*L, *ire*A, *iro*N, *iss*, *tir*	[109]
	Ivory Coast	Cattle	PCR	CTX-M, TEM, SHV	-	-	[82]
	Ghana	Poultry	WGS	CTX-M-15, SHV-12	-	-	[106]
	Tunisia	Poultry	PCR	CTX-M-1	-	-	[50]
		Cow		CTX-M-15			
		Sheep		CTX-M-1, CTX-M-15, TEM-1			
	Nigeria	Pig, sheep, cow, poultry	PCR	TEM, CTX-M-15, SHV	*aac*(6’)-*lb*	-	[51]
	Nigeria	Poultry	WGS	CTX-M, CTX-M-15, TEM	*tet*A, *sul*2, *mdf*A, *aph(3)*-Ib, *aph(6)*-Id, *dfr*A14	-	[89]
	Nigeria	Cow	Microarrays	CTX-M-15, CTX-M-9, TEM	*str*B, *sul*2	*hem*L, *iss*, *lpf*A	[98]
	Tanzania	Poultry	PCR	CTX-M, TEM, SHV	*aac(6)-Ib-cr*, *qnr*B, *qep*A	-	[85]
		Pig					
	Nigeria	Cow	WGS	CTX-M-14, CTX-M-15, CTX-M-55	*qnr*S1, *aph(6)*-Id, *aph(3)*-Ib, *aad*A2, *aad*A5, *aph(3)*-Id, *sul*2, *dfr*A14, *dfr*A17, *mdf*A, *tet*A	-	[83]
	Egypt	Poultry	PCR	CTX-M-9, TEM, OXA-2	-	-	[24]
	Algeria	Poultry	PCR	CTX-M-1	*tet*A, *sul*1	-	[110]
	Egypt	Poultry	PCR	CTX-M, SHV, TEM	-	-	[5]
		Cow					
	Nigeria	Cow, poultry, pig, sheep	qPCR, WGS	CTX-M-15, CTX-M-55, CTX-M-64, CTX-M-65, TEM-1	*str*A, *str*B, *aac3*-IId, *aad*A5, *sul*2, *sul*1, *dfr*A14, *dfr*A17, *mph*A	-	[84]
	Tunisia	Poultry	PCR	CTX-M-15, CTX-M-55, TEM, SHV-12	*aac(6’)*-Ib-cr, *sul*1, *tet*B	*fim*H, *fyu*A, *iut*A, *papGIII*	[80]
	Egypt	Poultry	PCR	TEM, SHV	-	-	[52]
America	Canada	Poultry	PCR	CTX-M-1	-	-	[107]
	Cuba	Poultry	PCR, microarrays	CTX-M-1, CTX-M-15	*tet*A, *tet*B, *mph*A, *sul*2, *dfr*A17, *str*A, *str*B	-	[90]
	USA	Cow, pig, poultry	PCR, WGS	CTX-M-1, CTX-M-9, TEM	*tet*A, *tet*B, *aph(6)*-Id, *sul*1, *sul*2, *sul*3, *str*A, *str*B, *aad*A2, *aph(3’)*-Ia	-	[22]
	USA	Cow	PCR	CTX-M-1, CTX-M-9	*mph*A, *qnr*B	-	[92]
			WGS	CTX-M-1, CTX-M-32, CTX-M-15, CTX-M-27, CTX-M-65	*aph(3’’)*-Ib, *aph(6)*-Id, *sul*1, *sul*2, *mph*A, *mdf*A, *tet*A, *flo*R		
	Mexico	Cow, poultry, pig, sheep	PCR	CTX-M, TEM	*tet*A, *tet*B, *aad*A1, *str*A, *str*B, *sul*1,2,3, *qnr*B	*hly*A	[86]
	Chile	Cow, poultry, pig, sheep	PCR	CTX-M, CTX-M-1, CTX-M-2, TEM, SHV	-	-	[20]
	Brazil	Poultry	Microarrays	CTX-M-1, CTX-M-2	-	-	[108]
			WGS	CTX-M-2, CTX-M-15			
	Brazil	Poultry	PCR	CTX-M, SHV	-	-	[91]
Asia	China	Pig	PCR, WGS	CTX-M, TEM- SHV, OXA-48, NDM	*qnr*S, *qnr*A, *aac(6’)*-Ib-cr, *qnr*B, *oqx*AB, *qnr*D, *qep*A	-	[57]
	Philippines	Poultry	PCR	CTX-M-1, CTX-M-15, CTX-M-25, CTX-M-2, CTX-M-8, CTX-M-9, TEM, SHV	-	-	[31]
	Thailand	Pig	PCR	CTX-M, TEM	-	-	[59]
			WGS	CTX-M-55, CTX-M-14, TEM-1B	*sul*1, *sul*2, *sul*3, *qnr*S1, *tet*A, *tet*D, *aad*A2, *aph(3’)*-Ia		
	Pakistan	Cow	PCR	CTX-M-15, TEM	-	-	[60]
		Poultry		CTX-M-15, CTX-M-55, TEM			
	Thailand	Poultry	PCR, WGS	CTX-M-15, CTX-M-55, CTX-M-14, CTX-M-27, CTX-M-65	-	-	[61].
	Malaysia	Cow	PCR	CTX-M, TEM	-	-	[62]
	China	Poultry	PCR	CTX-M-14, CTX-M-9, CTX-M-55, CTX-M-15, CTX.M-1, CTX-M-65, CTX-M-74, CTX-M-25, TEM, SHV	*qnr*S, *aac(6’)*-Ib-cr, *qnr*B, *qnr*A	*pap*C, *iuc*D, *iro*N, *iuc*D, *iss*, *iut*A, *tsh, irp*-2	[63]
	India	Pig	PCR	TEM, CTX-M, CMY	*tet*A, *tet*B, *sul*1, *sul*2, *aad*A, *dfr*Ia	-	[65]
	South Korea	Poultry	PCR	TEM-1, CTX-M-15, CTX-M-55, CTX-M-14, CTX-M-65	-	-	[39]
		Pig		TEM-1, CTX-M-3, CTX-M-15, CTX-M-55, CTX-M-14, CTX-M-65			
		Cow		CTX-M-15, CTX-M-55, CTX-M-65			
	India	Poultry	PCR	CTX-M, TEM, SHV	-	-	[65]
	India	Pig	PCR	CTX-M	-	-	[66]
	China	Poultry	PCR	TEM	-	-	[38]
		Pig		CTX-M, TEM			
	Indonesia	Poultry	PCR	CTX-M	-	-	[67]
	Pakistan	Poultry	PCR	CTX-M-1, CTX-M-9, TEM	-	-	[87]
	Thailand	Pig	PCR	CTX-M-55, CTX-M-14, CTX-M-15, CTX-M-9, OXA-140, SHV-12	-	-	[111]
	Korea	Pollo	PCR, WGS	CTX-M-55, CTX-M-14, CTX-M-65, CTX-M-1, CTX-M-27	*sul*1, *sul*2, *str*A, *str*B, *fos*A, *aac(3)*-IId, *mph*A	-	[112]
	Thailand	Pig	PCR	CTX-M-55, CTX-M-14, TEM	*mcr*-1	-	[100]
	Korea	Poultry	PCR	CTX-M-1, CTX-M-14, CTX-M-15, CTX-M-65, TEM-1	*dfr*A1, *aad*A1	-	[113]
	Vietnam	Pig	WGS	CTX-M-55, CTX-M-14, CTX-M-27, CTX-M-15, CTX-M-65, OXA-10	*mcr*-1, *mcr*-3, *qnr*S1, *qnr*B19, *aad*A1, *aph*(3’)-Ia, *aph*(6)-Id, *aac*(3)-IId, *aad*A2, *dfr*A12, *dfr*A14, *tet*A, *tet*M, *cml*A1, *flo*R, *mdf*A, *mef*A, *mef*B, *mph*A, *fos*A3, *aar*2, *aar*3, *sul*1, *sul*2, *chr*A, *mer*C, *mer*E, *mer*T	*tra*T, *omp*T, *sit*A, *lpf*A, *iss*, *ter*C, *tra*T, *chu*A, *fyu*A	[101]
	China	Cow	PCR	CTX-M, TEM- SHV	-	-	[23]
	Malaysia	Pig, poultry	PCR	CTX-M	-	-	[33]
	Indonesia	Pig	PCR	TEM	-	-	[74]
	China	Poultry	PCR	TEM, CTX-M, OXA, SHV	-	-	[75]
		Cow		TEM, OXA, SHV			
		Pig		TEM, OXA, CTX-M, SHV			
	India	Poultry	PCR, WGS	TEM, SHV, OXA, CTX-M-1, CTX-M-2, CTX-M-9	qnrS1, *dfr*A14, *sul*2, *aph*(3”)-Ib, *aph*(3’)-Ia, *aph*(6)-ld	*cib*, *ter*C, *tra*T	[76]
	Pakistan	Cow, poultry, sheep	PCR	CTX-M-1, CTX-M-9, CTX-M-2, TEM, SHV	*mcr*-1	-	[77]
	Thailand	Pig	PCR	CTX-M-55, CTX-M-14, TEM-1	-	-	[78]
	China	Sheep	WGS	CTX-M-55, CTX-M-15, CTX-M-14, CTX-M-65, CTX-M-17, TEM-1, TEM-150, TEM-235	*aph*(6’)-Id *aph*(3’)-Ia, *aph*(3’’)-Ib, *aad*A1, *aad*A2, *tet*A, *tet*B, *sul*1, *sul*2, *sul*3, *dfr*A12, *dfr*A14, *dfr*A17, *oqx*A, *oqx*B, *cml*A1, *cml*A5, *cat*A1, *cat*A2, *cat*A3, *flo*R, *mph*A, *erm*B, *fos*A, *mcr*-1	-	[79]
	Indonesia	Poultry	PCR	CTX-M, TEM	-	-	[94]
	Malaysia	Poultry	PCR	CTX-M, TEM	*mcr*-1	-	[88]
Europe	Spain	Cow, sheep	WGS	CTX-M-14, CTX-M-1, CTX-M-15, CTX-M-32 SHV-12, TEM-1B, TEM-190 OXA-1, OXA-10	*tet*A, *tet*B, *aac*(3)-IIa, *aac*(3)-IId, *qnr*S, *cml*1, *catA*1, *flo*R, *mph*A, *sul*1, *sul*2, *sul*3, *fos*A7	-	[36]
	Greek	Cow, pig	Microarrays	CTX-M-15, TEM	*aad*A1, *aph*A, *str*A, *str*B, *qnr*S, *sul*1, *sul*2, *sul*3, *dfr*A7, *dfr*A1, *mph*	-	[68]
	Portugal	Sheep	PCR	CTX-M-15, CTX-M-32, CTX-M-1, CTX-M-14, CTX-M-98, SHV-12	*aac(6’)*-Ib-cr, *qnr*S, *aac(3’)*-II, *tet*A, *tet*B, *sul*1, *sul*2, *sul*3	-	[70]
	Denmark, France, Germany, Hungary, Poland, Spain, Netherlands, UK	Poultry	WGS	CTX-M-1, CTX-M-14, SHV-12, TEM-52	*aad*A1, *aad*A2, *aad*A5, *tet*A, *tetB, dfr*A1, *dfr*A7, *sul*1, *sul*2, *sul*3, *cat*A1, *flo*R, *mph*A, *mph*B	*ent*ABCDEFS, *fim*ABCDEFGHI, *csg*ABCDEFG	[71]
		Cow		CTX-M-1, CTX-M-2, SHV-12, TEM-52			
		Pig		CTX-M-1, CTX-M-15			
	Bosnia	Poultry	Microarrays	CTX-M-1, CTX-M-15, TEM, SHV	-	-	[72]
	Latvia	Pig	PCR	CTX-M, TEM, SHV	-	-	[4]
	France	Cow, pig, poultry	PCR	CTX-M-1, CTX-M-15, TEM-1B, TEM-1C	*tet*A	-	[73]
	Italy	Cow	PCR	CTX-M-1, CTX-M-9	*mcr*-1, *mcr*-3	-	[21]
		Pig		CTX-M-1, CTX-M-9	*mcr*-1, *mcr*-4		
		Poultry		CTX-M-1, CTX-M-2, CTX-M-9, SHV-12	*mcr*-1		
	Greece	Pig	Microarrays	CTX-M-15, CTX-M-9, CTX-M-8, SHV, TEM, OXA-1, OXA-60	*aad*A1, *aad*A2, *aad*A4, *aac*(6’)-Ib, *qnr*S, *qnr*A, *qnr*B, *sul*1, *sul*2, *sul*3, *dfr*A1, *dfr*A5, *dfr*A7, *dfr*A12, *mcr*-1, *mcr*-2, *mcr*-4, *mcr*-8, *mph, oqx*A, *oqx*B	-	[103]
	Macedonia	Cow	PCR	CTX-M, SHV, TEM, OXA-1	-	-	[93]
Oceania	Australia	Pig	RT-PCR	CTX-M-1, TEM-1B	*aad*A5, *dfr*A17, *dfr*A5, *sul*2, *tet*A, *str*A, *str*B	*eae*, *ehx*A, *paa*	[19]

## Data Availability

Not applicable.
